# Response to Dr. Moya’s Comments to Article by Thoene M et al., *Nutrients* 2016, *8*, 451

**DOI:** 10.3390/nu8120822

**Published:** 2016-12-16

**Authors:** Melissa Thoene, Ann Anderson-Berry

**Affiliations:** 1 Newborn Intensive Care Unit, Nebraska Medicine, 981200 Nebraska Medical Center, Omaha, NE 68198, USA; 2 Department of Pediatrics, University of Nebraska Medical Center, 981205 Nebraska Medical Center, Omaha, NE 68198-1205, USA; alanders@unmc.edu

**Keywords:** human milk, fortifier, premature infant, enteral nutrition, growth, acidosis, necrotizing enterocolitis

## Abstract

This reply is a supplementary addition to our previous article entitled “Comparison of a Powdered, Acidified Liquid, and Non-Acidified Liquid Human Milk Fortifier on Clinical Outcomes in Premature Infants” as published in *Nutrients* in July 2016. It provides a response to a comment made by Dr. Fernando Moya to this original article, so the purpose of this is to compare and contrast various perspectives between researchers conducting nutrition research in the preterm infant population. It specifically focuses on human milk fortification and subsequent outcomes.

To the editor:

We have read the comments by Dr. Fernando Moya to our article entitled “Comparison of a Powdered, Acidified Liquid, and Non-Acidified Liquid Human Milk Fortifier on Clinical Outcomes in Premature Infants” as published in *Nutrients* in July 2016 [1]. We value the input of others participating in research among the preterm infant population.

Moya reiterates the importance of understanding population demographics and research methods between studies to accurately compare and contrast. Agreeably, this is an imperative step when comparing the results of our study with those of Moya et al. [1,2]. In the instance of our study, our baseline criterion was to include infants <2000 grams whereas Moya et al. included infants ≤1250 grams [1,2]. The purpose of our wider inclusion criteria was to assess outcomes for a larger demographic population of low birth weight infants who also require essential human milk fortification. We chose not to exclude infants based on criteria like low Apgar score or high ventilatory needs as this would eliminate a substantial percent of our high acuity population, and would therefore be less reflective of practice in a true Newborn Intensive Care Unit (NICU) setting. Additionally, we chose not to report descriptive statistics like mode of delivery, Apgar score, etc., as this makes limited difference on our clinical decisions for nutrition management. Infants in the study by Moya et al. were reported to be born at younger gestational ages and lower birth weights compared to those in our study [1,2]. However, this difference is attributed to our inclusion of infants born with weights up to 2000 grams [1].

Despite infants in the Moya et al. study being born at younger gestational ages and with lower birth weights, it must be noted that the study period for this population did not begin until these infants were around 18 days of life [1,2]. Therefore, their carbon dioxide levels reported at study day 14 and 28 were reported around day of life 32 and 46. This contrasts our study which reported levels after day of life 14 and 30, meaning infants in Moya et al. were assessed at older days of life [1,2]. Another key difference is that study infants in Moya et al. began the study period at the start of human milk fortification when enteral feedings reached 80 mL/kg/day [1,2]. While not reported, it may be anticipated that infants received remaining nutrition from parenteral nutrition, which in turn would allow for initial closer management of acid-base balance. In contrasting review, at least half of our study infants receiving the AL-HMF had achieved full enteral feedings at median day of life 10, well before when Moya et al.’s infants even began receiving human milk fortifier [1,2]. We cannot attribute our finding of lower carbon dioxide levels to preterm infants having poor metabolic adaptations in early life, as infants in our study receiving the P-HMF and NAL-HMF achieved full enteral feedings at similar days of life and experienced normal levels [1].

We reported no incidence rate of metabolic acidosis, but our institutions reference range for neonatal carbon dioxide level is 22–32 mmol/L. Our results clearly demonstrate that the median carbon dioxide level in our AL-HMF group remained below this reference range [1,2]. This is similar in findings to Cibulskis et al. who reported a 57% rate of metabolic acidosis in infants receiving AL-HMF compared to 10% for those receiving a powdered HMF [3].

While studies by Moya et al. and Cibulskis and Armbrecht demonstrated similar growth in the AL-HMF group compared to other fortifiers, our infants experienced a significant reduction in growth velocity despite receiving higher calories and protein [1,2,3]. Additionally, we would gently correct Dr. Moya when comparing growth results from our study and the Cibulskis study. When evaluated in grams/kg/day (the gold standard of growth evaluation in this population), our powdered HMF group grew at 15.37 grams/kg/day respectively compared to 14.7 g/day from the Cibulskis powdered group [1,3]. Again, comparing groups recruited from different protocols with different time points is relatively unhelpful. We also see that Cibulskis et al. reported a higher incidence of infants having milk fortification discontinued (62% vs. 18%, *p* < 0.001) for intolerance or acidosis, which knowingly hinders nutrition delivered [3]. Furthermore, it is important to consider the results by Erickson et al. who noted a reduction in human milk white blood cells, lipase activity, and protein content when acidified [4]. While milk fortification is necessary, it should remain a priority to preserve mother’s milk as best able to enhance the benefits to these fragile infants.

Our study reported NEC rates based on diagnosis using Bell’s staging criteria, so were not based on non-specific feeding intolerance as Moya implies. While our findings were statistically significant, we did report that NEC was not powered as a primary outcome for this study [1]. Our policy is to utilize donor milk as needed based on the mother’s milk supply until 14 days of life, at which point infants would be slowly transitioned to preterm infant formula over 2–3 days. No infants were diagnosed with NEC prior to transitioning to >50% of feedings as formula. Additionally, our baseline rates of NEC have historically remained low [5].

Growth cannot be directly compared between our study and Moya et al. [1,2]. Unique to our study is that growth was measured while infants were on full enteral feedings and for as long as they received ≥50% of feedings as fortified human milk. Moya et al. reports growth throughout a designated study period (28 days), in which infants were not on full enteral feedings at the start of the study period [1,2]. Mean day of life to achieving full enteral feedings was not reported. It is unclear if infants received supplemental parenteral nutrition until full enteral feedings were attained, which has the potential to influence growth outcomes. We reported the number of infants requiring caloric density feedings >24 calories-per-ounce and average daily delivered calories and protein per kilogram, which is not reported by Moya et al. outside of a few infants receiving additional protein supplementation [1,2].

While our study was not a randomized, controlled trial, it has merit given that the data was retrospectively collected based on concerning clinical findings. While we agree that the neonatology community may benefit from a randomized, controlled trial, the value of having a more inclusive population of infants who require human milk fortification should be considered. It would also be important to conduct the study where feeding practices are standardized and infants are able to achieve full enteral feedings more quickly than at several weeks of life. Considering the increasing number of studies consistently reporting significant metabolic acidosis as a side effect of this nutrition intervention, it is also important to ask ourselves if we as a medical community have equipoise to enroll our most fragile patient population in such a study.
